# Analysis of Genome DNA Methylation at Inherited Coat Color Dilutions of Rex Rabbits

**DOI:** 10.3389/fgene.2020.603528

**Published:** 2021-01-21

**Authors:** Yang Chen, Shuaishuai Hu, Ming Liu, Bohao Zhao, Naisu Yang, Jiali Li, Qiuran Chen, Juan Zhou, Guolian Bao, Xinsheng Wu

**Affiliations:** ^1^College of Animal Science and Technology, Yangzhou University, Yangzhou, China; ^2^Joint International Research Laboratory of Agriculture & Agri-Product Safety, Yangzhou University, Yangzhou, China; ^3^Animal Husbandry and Veterinary Research Institute Zhejiang Academy of Agricultural Sciences, Hangzhou, China

**Keywords:** DNA methylation, WGBS, coat-color dilution, rabbit, pigmentation

## Abstract

**Background:** The dilution of color in rabbits is associated with many different genetic mechanisms that form different color groups. A number of previous studies have revealed potential regulatory mechanisms by which epigenetics regulate pigmentation. However, the genome-wide DNA methylation involved in animal coat color dilution remains unknown.

**Results:** We compared genome-wide DNA methylation profiles in Rex rabbit hair follicles in a Chinchilla group (Ch) and a diluted Chinchilla group (DCh) through whole-genome bisulfite sequencing (WGBS). Approximately 3.5% of the cytosine sites were methylated in both groups, of which the CG methylation type was in greatest abundance. In total, we identified 126,405 differentially methylated regions (DMRs) between the two groups, corresponding to 11,459 DMR-associated genes (DMGs). Gene ontology and Kyoto Encyclopedia of Genes and Genomes pathway analysis revealed that these DMGs were principally involved in developmental pigmentation and Wnt signaling pathways. In addition, two DMRs were randomly selected to verify that the WGBS data were reliable using bisulfite sequencing PCR, and seven DMGs were analyzed to establish the relationship between the level of DNA methylation and mRNA expression using qRT-PCR. Due to the limitation of small sample size, replication of the results with a larger sample size would be important in future studies.

**Conclusion:** These findings provide evidence that there is an association between inherited color dilution and DNA methylation alterations in hair follicles, greatly contributing to our understanding of the epigenetic regulation of rabbit pigmentation.

## Background

**Figure 1:**
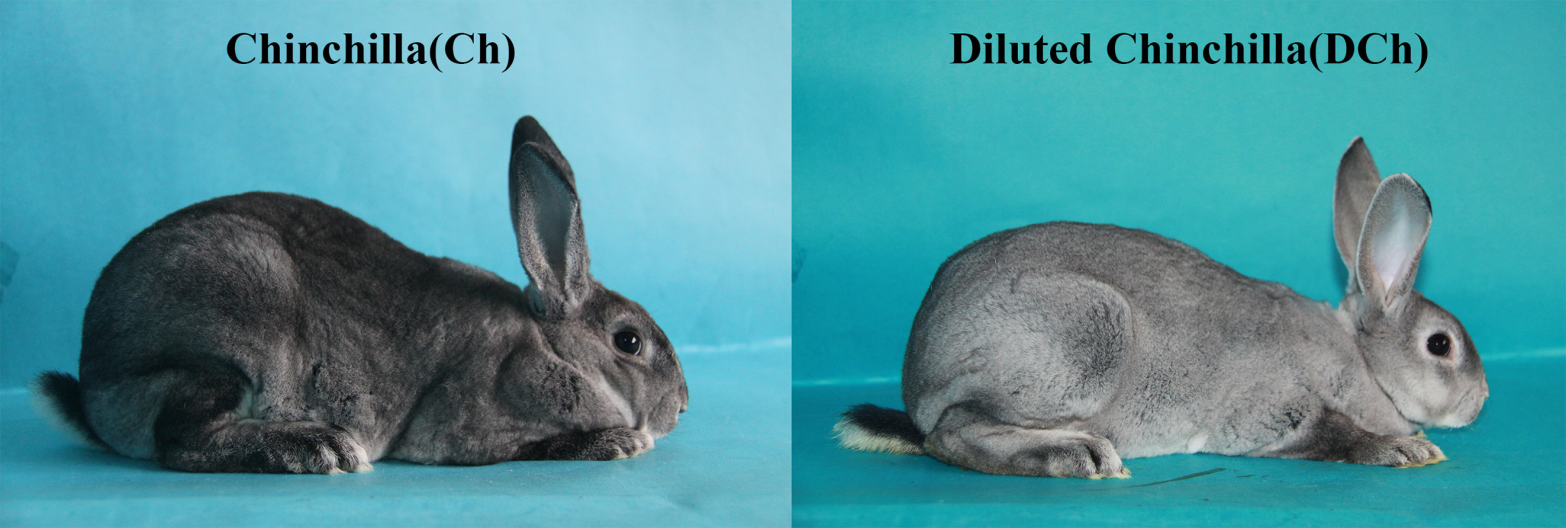
Individual phenotypic appearance of Chinchilla and diluted Chinchilla rabbits.

During the long-term domestication of rabbits (*Oryctolagus cuniculus*), different coat colors and color patterns have been selected and bred; specific strains and breeds are usually referred to by their color. The Chinchilla is among the more striking breeds, with a characteristic coat pattern being displayed in interphase dark blue–gray. American Chinchilla rabbit colors include light, medium, and dark shades. Light and medium Chinchilla rabbits exhibit a matte dilution relative to dark. Here we present two genetically stable Chinchilla rabbits, one being the standard Chinchilla and the other a diluted Chinchilla (Figure 1). The under ring color of the standard Chinchilla coat is dark and blue, the middle ring is pearl gray, and the top edge is a very narrow black band. In contrast, the under and top ring colors of the diluted Chinchilla coat are lighter. Two exon skipping and a frameshift mutation within the melanophilin (MLPH) gene are associated with coat color dilution in rabbits (Lehner et al., 2013; Fontanesi et al., 2014). However, these previously reported mutations associated with coat color dilution in rabbits were not identified in the Chinchilla populations investigated here (Li et al., 2020). Moreover, the mutations in exons of the MLPH gene were detected in the DCh group (Supplementary Table 1). We speculated that variants other than the reported ones may be associated with coat color dilution in the DCh group under study. The mechanism of hair color formation needs further investigations.

Coat color dilution in animals is regulated by eumelanin and pheomelanin. Dilution of eumelanin in black and brown coat colors can produce blue and cream–brown, while dilution of pheomelanin in a yellow coat color can result in a cream–yellow coat. In addition to rabbits, there are several known dilution phenotypes that are identical and favored by breeding in different species, including cats (Prieur and Collier, 1981), chicken (Vaez et al., 2008), quails (Minvielle et al., 2010), mice (Russell, 1946), foxes (Bradbury and Fabricant, 1988), and mink (Anistoroaei and Christensen, 2007). Dilution of coat color in dogs may be accompanied by alopecia (Philipp et al., 2005; Perego et al., 2009). At present, dilution of color is associated with several different genetic mechanisms resulting in distinct color groups. The mechanisms exist in numerous species and varieties but are not consistent in the majority of cases.

It has been documented that modification by methylation is one cause of variations in mammalian coat color. When pregnant yellow agouti (Avy) mice were supplemented with a methyl donor component, the offspring's coat color shifted to gray, shown to result from methylation of the retroviral long terminal repeat promoter (Waterland and Jirtle, 2003). Folic acid or the phytoestrogen genistein supplied by maternal nutrition can counteract DNA hypomethylation and transform coat color distribution (Dolinoy et al., 2006). The dilution of hair color in the somatic cell-cloned pig is directly caused by methylation of the promoter region of the KIT gene (Hwang et al., 2009). A scrutiny of the studies described above suggests that modifications by methylation are important in genetic studies of animal hair color.

The present study aimed to identify the genome-wide DNA methylation patterns in Chinchilla and diluted Chinchilla rabbit hair follicles and thus the candidate genes responsible for coat color dilutions. This study provides the basis for speculation about the epigenetic mechanisms that lead to color dilution in the coats of Rex rabbits and provides a reference for the development of appropriate breeding programs.

## Methods

### Animals and Tissue Collection

Five-month-old female Rex rabbits were provided by Zhejiang Yuyao Xinnong Rabbit Industry Co., Ltd., including Chinchilla (Ch, *n* = 3) and diluted Chinchilla (DCh, *n* = 3) varieties. All rabbits were fed a commercial diet (17% crude protein, 15% fiber, 3% fat, 10.4 MJ/kg digestible energy, 1% calcium, and 0.5% phosphorus). Food and water were provided *ad libitum*. The does were housed in an individual cage (60 × 40 × 35 cm) under natural lighting and controlled ventilation. The rabbits were anesthetized by an intra-articular injection of 0.7% sodium pentobarbital. Hair follicle samples were harvested from the back of each rabbit, immediately snap-frozen in liquid nitrogen, and then stored at −80°C until required for use. Iodophor was administered to tissue explant sites to avoid bacterial infection. The rabbits have made a full recovery from the operation and were free to move in an hour.

### Total DNA Extraction and DNA Library Construction

Genomic DNA was extracted from the hair follicle samples of Ch and DCh rabbits using a genomic DNA kit (TIANamp, China). DNA purity and concentration were measured using a NanoPhotometer® spectrophotometer (Implen, CA, USA) and a Qubit® DNA assay kit in a Qubit® 2.0 fluorometer (Life Technologies, CA, USA), respectively.

Genomic DNA was sonicated with Covaris S220 to obtain a 200–300-bp fragment with end repair and adenylation. A cytosine-methylated barcode was ligated to the sonicated DNA, and these DNA fragments were treated twice with bisulfite in accordance with the instructions of an EZ DNA Methylation-Gold^TM^ kit. Subsequently, the single-stranded DNA fragment obtained in this way was amplified by PCR using KAPA HiFi Hot-Start Uracil+ ReadyMix (2×). Concentration was determined using a Qubit® 2.0 Fluorometer (Life Technologies, CA, USA), and the size of the insert was measured using an Agilent Bioanalyzer 2100 system. Cluster generation was accomplished using a cBot Cluster Generation System using a TruSeq PE cluster kit v3-cBot-HS (Illumia). The library was then sequenced using an Illumina Hiseq 2500 platform, and 125-bp paired-end reads were generated.

### Mapping Reads to Known Genome

The raw reads were filtered to obtain clean reads stored in FASTQ, by removing adapters, Ns, and low-quality reads. An alignment analysis of the reference genome (OryCun 2.0) was performed, and methylation data were extracted using Bismark software (version 0.16.1) (Krueger and Andrews, 2011). Both C-T and G-to-A (reverse complementation) transformations on the sequenced results from the reference genome were ascertained for pairwise alignment using bowtie2 (Langmead and Salzberg, 2012). The results were visualized in bigWig format using an Integrative Genomics Viewer browser. The non-conversion rate of bisulfite was calculated using the percentage of cytosine sequenced at the cytosine reference site. Based on coverage ≥5× and false discovery rate <0.05, we performed a binomial distribution test for each C site to identify methylated Cs (Gifford et al., 2013; Habibi et al., 2013).

### Estimating Methylation Levels and the Identification of DMRs

To confirm methylated sites, we modeled the sum $s_{i,j}^{+}$ of methylated counts as a binomial (Bin) random variable with a methylation rate: $r_{i,j,}s_{i,j}^{+\sim }BIn(s_{i,j}^{+}+s_{i,j}^{-},r_{i,j})$.

The sequence was then divided into multiple bins with a size of 10 kb to calculate the methylation level. We calculated the sum of methylated and non-methylated read counts in each window. The methylation level (ML) for each window or C site indicates the proportion of methylated Cs, defined as:

(1) $$\begin{array}{r} \text{ML}\left(\text{C}\right)=\frac{\text{reads}\left(\text{mC}\right)}{\text{reads}\left(\text{mC}\right)+\text{reads}\left(\text{C}\right)} \end{array}$$

Differentially methylated regions (DMRs) were identified using Bsseq software with a sliding window approach. The window was set to 1,000 bp and the step length to 100 bp.

### Gene Ontology and KEGG Pathway Analysis of DMGs

DMR-associated genes (DMGs) were analyzed based on DMRs that overlapped gene functional regions, such as promoter, 5′-UTR, exon, intron, and 3′-UTR regions, with at least 1 bp. The DMGs were screened and annotated by gene ontology (GO) and Kyoto Encyclopedia of Genes and Genomes (KEGG) enrichment analysis. GO enrichment analysis was achieved using the GOseq R software package (Young et al., 2010), in which GO items with a corrected *P*-value < 0.05 were considered as significantly enriched. KEGG can be used to analyze the advanced functions and biological systems (such as cells, organisms, and ecosystems) at a molecular level (http://www.genome.jp/kegg/) (Kanehisa et al., 2008). KOBAS software was used to analyze the statistical enrichment of DMGs in KEGG pathways (Mao et al., 2005).

### Bisulfite Sequencing PCR

Five hundred nanograms of genomic DNA was modified and purified using an EpiTect Fast DNA bisulfite kit (Qiagen, Germany). Converted DNA was stored at −20°C until required for use. Bisulfite sequencing PCR (BSP) primers were designed using Meth-Primer software (http://www.urogene.org/methprimer) as presented in Supplementary Table 2. A 50-ng quantity of converted DNA was used in a 50-μl reaction system. These PCR products were cloned into a pMD19-T vector (Takara, Dalian, China). Ten clones per sample were sequenced. The methylation levels were evaluated by calculating the percentage of converted cytosines to the total number of cytosines. The BSP results were aligned using MegAlign software, and an analysis was conducted at the MSR website (http://www.msrcall.com/MSRcalcalate.aspx).

### Quantitative Real-Time PCR

Quantitative real-time PCR (qRT-PCR) was conducted using ChamQ^TM^ SYBR® qPCR Master Mix (Vazyme) on an Applied Biosystems® QuantStudio® 5 Real-Time PCR system using the following parameters: pre-denaturation stage−95°C for 30 s; PCR reaction−95°C for 10 s, 60°C for 30 s (40 cycles); and dissolution at 95°C for 15 s, 60°C for 1 min and 95°C for 15 s. The RT-PCR primer sequences are presented in Supplementary Table 3. The results were normalized to GAPDH expression. The relative expression of DMGs was calculated using the ^ΔΔ^Ct method = 2 ^(ΔCt experimental−ΔCt control)^ = 2^−ΔΔCt^.

### Statistical Analysis

For BSP and qRT-PCR, each experiment was repeated at least three times, and the statistical significance was determined using an independent-sample *T*-test. All results are presented as means ± SD at two levels of significance, ^*^P < 0.05 and ^**^P < 0.01.

## Results

### DNA Methylation Mapping, Patterns, and Sequence Preference Analysis

**Figure 2:**
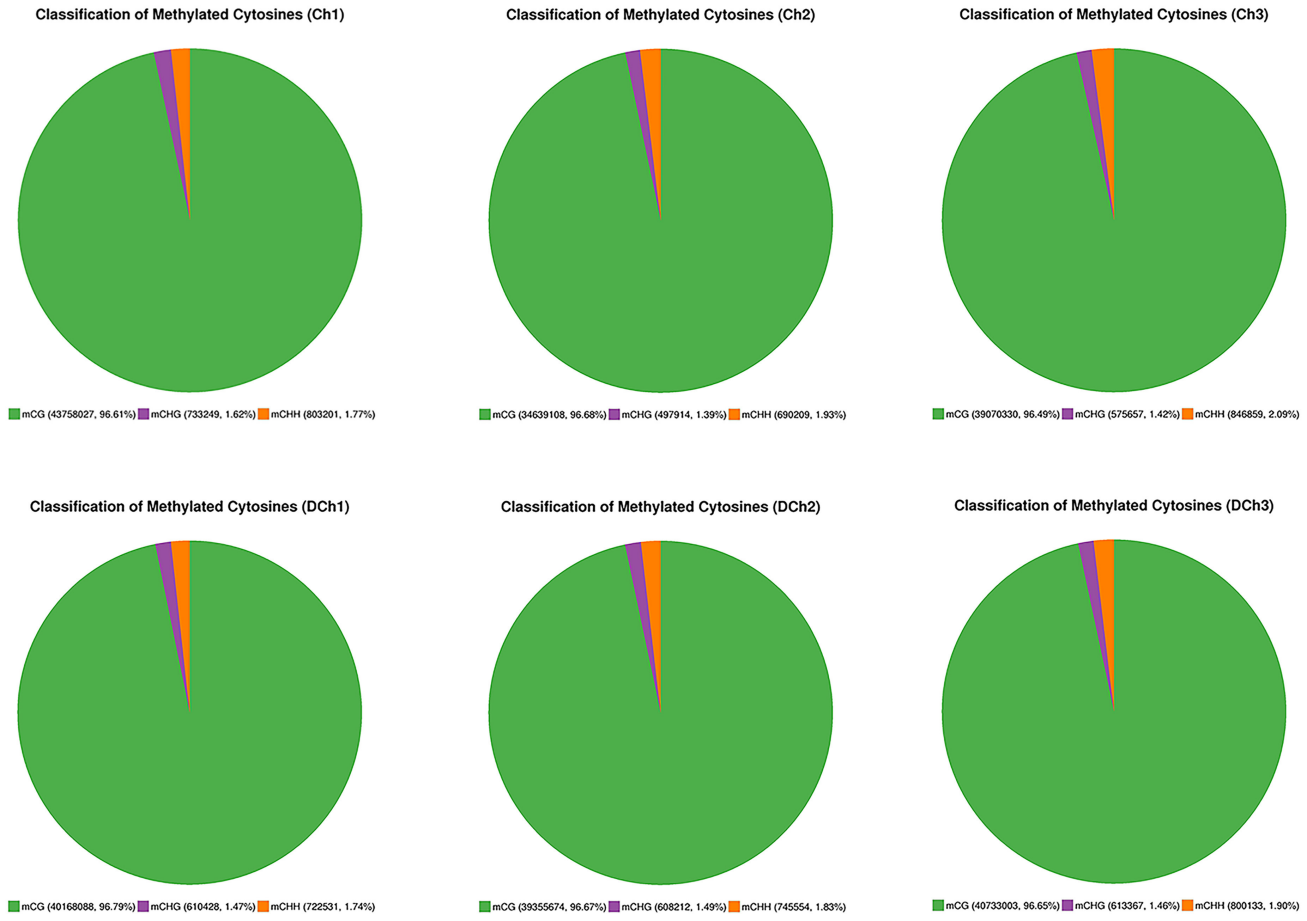
Distribution of mCG, mGHG, and mCHH in all methylated cytosine residues.

A total of 105.40 ± 2.26 and 102.36 ± 3.87 G raw bases were generated from the Ch and DCh groups, respectively. After data filtering, more than 320 million clean reads were obtained in each group, which were detected in all chromosomal regions. The ratio of the number of bases (≥10 × the sequencing depth) to the genome per sample was more than 80%. The mapping rate ranged from 67.77 to 70.89%, suggesting that the data can be utilized in a subsequent analysis (Table 1). In both groups, ~3.5% of all genomic C sites were methylated. Methylation was found in three sequence contexts in similar proportions in each group, namely, CG, CHG, and CHH (where H was A, C, or T). The CG type was the most abundant form at 96.49–96.79%. The overall genome-wide methylated cytosine levels of CHG and CHH were <3.60% in both of the two groups of rabbits (Figure 2).

**Table 1:** Read quality and genome coverage.

**Sample name**	**Ch1**	**Ch2**	**Ch3**	**DCh1**	**DCh2**	**DCh3**
Clean base (Gb)	95.79	97.13	92.26	90.44	90.59	97.12
Clean reads^a^	343,286,864	347,831,906	330,086,043	323,729,478	321,049,987	347,277,157
Mapping rate^b^ (%)	68.43	70.89	70.76	67.77	68.67	69.18
Bisulfite conversion rate^c^ (%)	99.25	99.27	99.25	99.16	99.15	99.24
mC percent^d^ (%)	3.98	3.14	3.55	3.64	3.57	3.70
sites_numCovg1^e^ (%)	93.97	93.66	93.91	93.92	93.83	93.99
sites_numCovg5^e^ (%)	91.16	88.99	90.38	90.71	90.24	90.77
sites_numCovg10^e^ (%)	85.52	80.73	83.19	83.74	81.99	83.66

a *Trimmed, filtered sequencing data and subsequent bioinformatics analysis were based on clean reads*.

b *Percentage of the matched clean reads to the total clean reads*.

c *The ratio of conversion of C into T by bisulfite*.

d *Percentage of methylated cytosine in the whole genome as a percentage of all cytosines*.

e *The ratio of the number of bases (≥1×/5×/10× the sequencing depth) to the genome*.

### DNA Methylation Levels in Different Genomic Functional Regions

**Figure 3:**
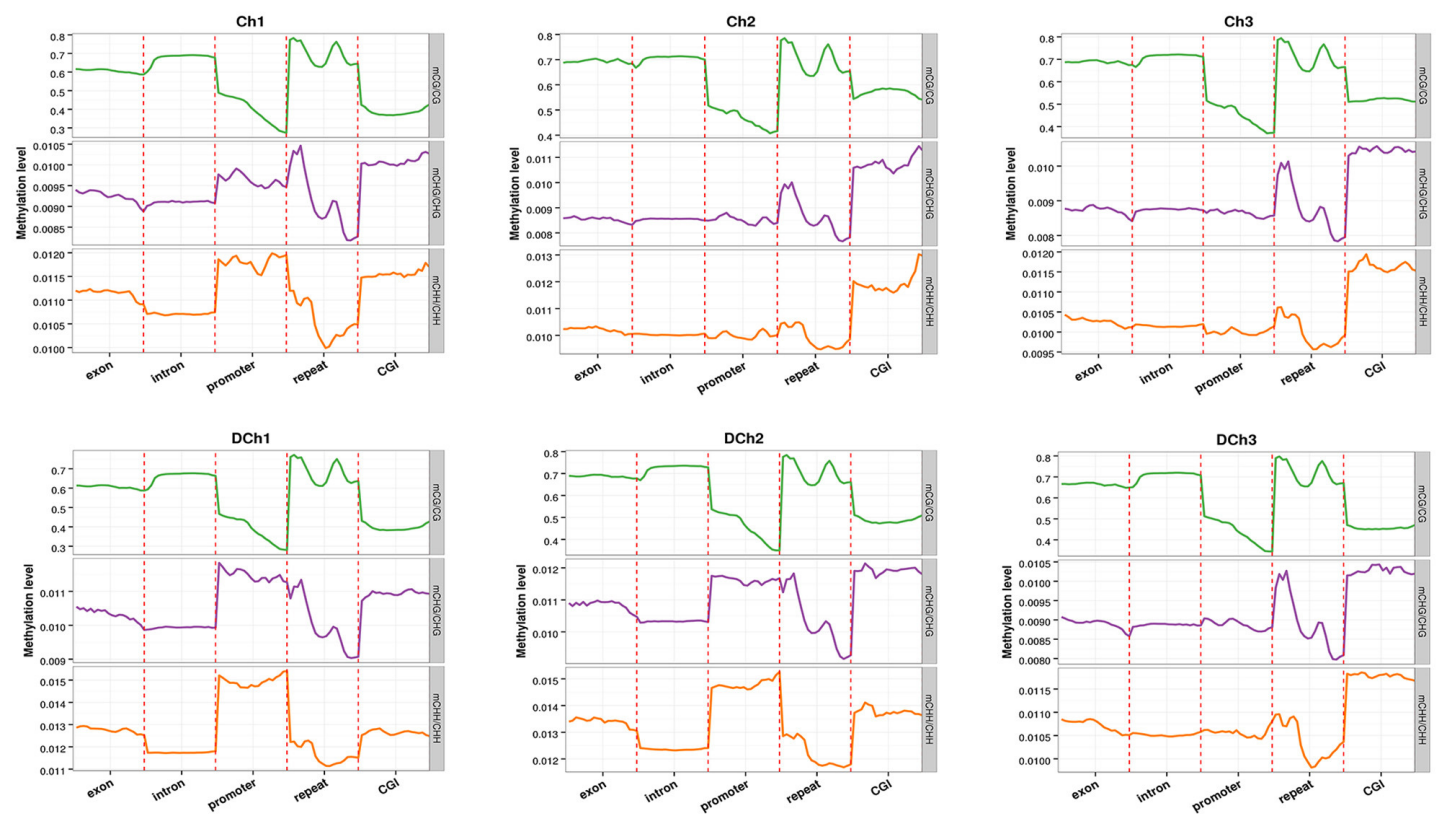
DNA methylation levels in different genomic elements. The abscissa represents the different genomic elements and the ordinate the methylation level. The functional regions of each gene were divided equally into 20 bins and then averaged at the C site level of the corresponding bin. Different colors represent different sequence contexts (CG, CHG, or CHH).

To explore the role of methylation in transcriptional regulation, we analyzed the DNA methylation levels in different genomic functional regions (e.g., promoter, exon, intron, repeat, etc., where the promoter region was 2 kb upstream of the transcription start site) in different sequence contexts as shown in Figure 3. For the CG type, similar methylation levels were observed in each functional element for the mC in six samples. The DNA methylation levels were greatest in repeats, followed by exon, intron, and CGI, with the promoter region being the lowest. For CHG and CHH types, the CGI regions and promoter exhibited a different methylation status. The methylation levels of the functional elements of the other genomes were consistent.

### Identification of Differentially Methylated Regions Between Ch and DCh

**Figure 4:**
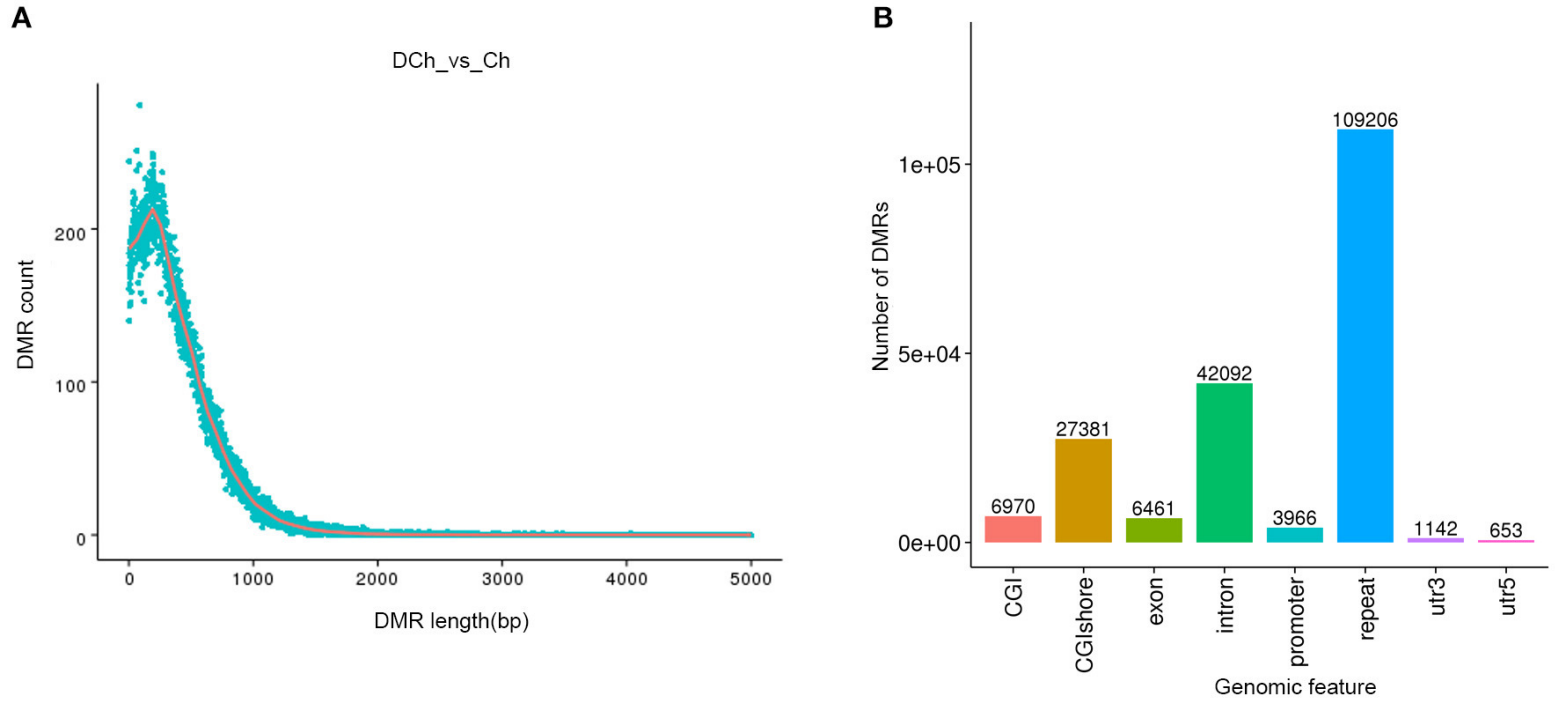
Identification and structural annotation of differentially methylated regions (DMRs). **(A)** Distribution of DMR length. The abscissa represents DMR length; the ordinate displays the DMR frequency for each corresponding length. **(B)** Functional region distribution of the DMRs. The abscissa represents different functional regions, with the ordinate indicating the number of DMRs in different functional regions.

After annotation into genetic functional regions, a total of 126,405 DMRs were identified (Supplementary Table 4). The distribution of lengths of the DMRs was calculated. The length of 71.12% of the DMRs was found to be ≤500 bp (Figure 4A). The DMRs were aligned to different genomic elements, mostly in the repeat region, followed by the intron and CGI shore (Figure 4B). A total of 11,459 DMGs were identified, of which 9,410 were differentially hyper-methylated in the DCh rabbits and 2,049 in the Ch rabbits. Among them, 3,850 DMRs that matched the promoter region were detected, and these DMRs were annotated in the promoter region of 3,581 genes. More detailed DMR results are presented in Supplementary Table 5.

### Functional Enrichment Analysis of DMGs

To explore the function in these methylated regions based on coat color traits, GO and KEGG pathway analyses were conducted to annotate the 11,459 DMGs. The GO analysis indicated that the DMGs were involved in biological processes important for the formation of coat color, including developmental pigmentation (GO:0048066), pigmentation (GO:0043473), positive regulation of MAPK cascade (GO:0043410), regulation of gene expression, epigenetic (GO:0040029), regulation of Wnt signaling pathway (GO:0030111), etc. The KEGG analysis indicated that the DMGs were enriched in the PI3K-Akt signaling pathway, mTOR signaling pathway, melanoma, MAPK signaling pathway, melanogenesis, Wnt signaling pathway, Notch signaling pathway, etc. More detailed results of the GO and KEGG analyses are presented in Supplementary Tables 6, 7.

### Verification of DMGs by Bisulfite Treatment and qRT-PCR

**Figure 5:**
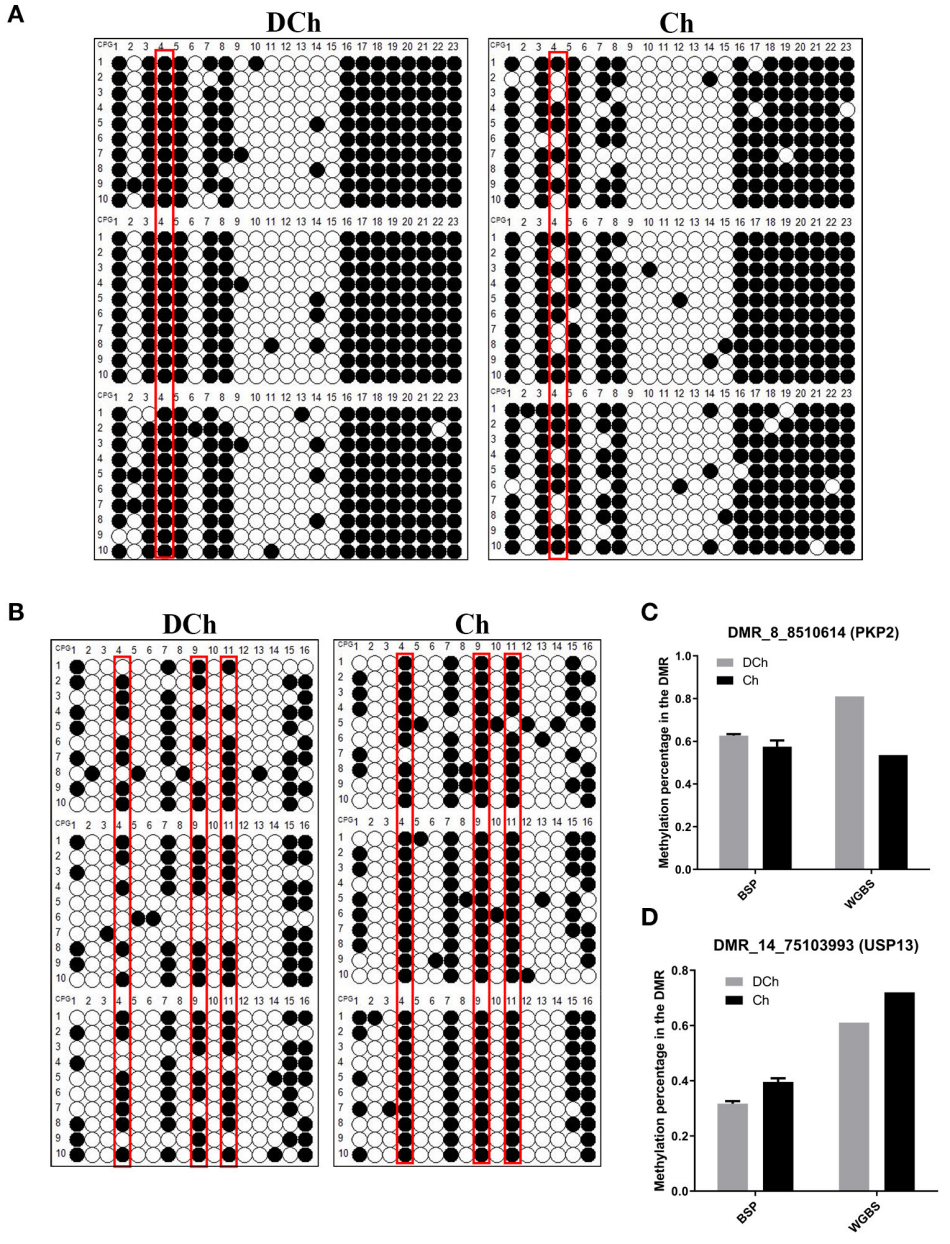
Verification of DMR-associated genes (DMGs) by bisulfite sequencing PCR (BSP). **(A)** BSP analysis in the DMR_8_8510614 (PKP2) region. The black and white circles indicate methylated and non-methylated C sites, respectively. Each row represents a separate clone. Red boxes indicate significant methylation sites as identified using Fisher's exact test. **(B)** BSP analysis in the DMR_14_75103993 (USP13) region. **(C)** Comparison of the degree of methylation of DMR_8_8510614 (PKP2) between BSP and whole-genome bisulfite sequencing (WGBS). For BSP, the ordinate represents the mean methylation rate of three samples in each group, while for WGBS, it represents the methylation level of the mean normalized DMR. The results represent means ± SD at two levels of significance. **(D)** Comparison of the extent of methylation of DMR_14_75103993 (USP13) between BSP and WGBS.

Two DMRs (one hypo-DMR and one hyper-DMR) from the DCh group were randomly selected to verify the reliability of the WGBS data by BSP, namely, DMR_8_8510614 (PKP2) and DMR_14_75103993 (USP13). The DCh group exhibited hypermethylation of PKP2, compared with the degree of methylation observed in the Ch group (*P* < 0.05). Conversely, DCh exhibited hypo-methylation of USP13, compared with the extent of methylation in Ch (*P* < 0.05). The methylation analysis of two DMRs was consistent with BSP and WGBS, indicating that the WGBS data in this study were reliable (Figure 5).

**Figure 6:**
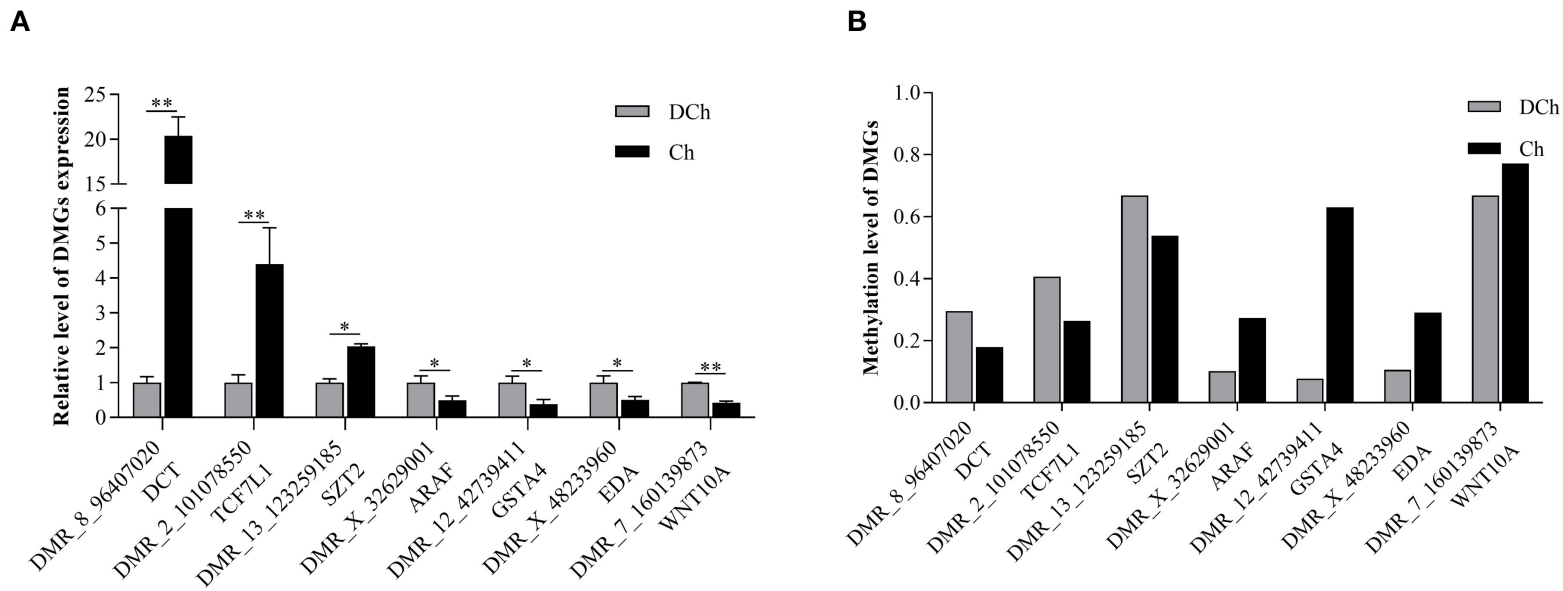
Verification of DMR-associated genes (DMGs) by qRT-PCR. **(A)** mRNA expression levels of seven DMGs by qRT-PCR. **(B)** DNA methylation levels of seven DMGs based on whole-genome bisulfite sequencing data *P < 0.05 and **P < 0.01.

To further validate the methylation status and expression of the DMGs, seven that were annotated in the promoter region, as identified from the results of WGBS, were selected for quantification by qRT-PCR. As shown in Figure 6, the mRNA expression of DCT, TCF7L1, and SZT2, respectively, were significantly lower, and those of ARAF, GSTA4, EDA, and WNT10A were significantly higher in the DCh compared with those of the Ch group (*P* < 0.05). The results were consistent with the negative regulatory relationship between DNA methylation levels and mRNA gene expression.

## Discussion

DNA methylation plays an important role in genomic stability, gene activation, X-chromosome inactivation, and other processes such as epigenetic regulatory mechanisms that participate in numerous life processes, including pigmentation (Morgan et al., 1999; Waterland and Jirtle, 2003; Dolinoy et al., 2006; Hwang et al., 2009). Based on an updated literature search, genomic regions targeted by the environment that escape epigenetic reprogramming could undergo epigenetic inheritance (Radford, 2018). In the present study, we presented two genetically stable Chinchilla rabbits breeds: one is the standard Chinchilla and the other is the diluted Chinchilla. The formation of coat color dilution phenotypes may be influenced by temperature and climate on hair follicle growth and color intensity initially. In order to explore the role of epigenetics in coat color inheritance, we compared the genome-wide methylation patterns in the DCh and Ch rabbit hair follicles to identify DMRs related to the dilution of coat color. In both groups, the proportion and the type of methylated cytosine site were similar to those of other species, such as humans, pigs, sheep, and chicken (Lister et al., 2011; Li et al., 2015; Hao et al., 2016; Zhang et al., 2017). This indicates that the DNA methylation patterns of different species have similarities and are conserved.

We identified 126,405 DMRs, and 11,459 genes related to these DMRs were obtained. In order to confirm the reliability of WGBS, we used the BSP method to detect the methylation levels of DMR_8_8510614 (PKP2) and DMR_14_75103993 (USP13), the results of which were consistent with the sequencing data. Generally, DNA methylation occurring within a promoter region or close to a transcription start site negatively regulates gene expression (Klose and Bird, 2006; Jones, 2012). Among the 126,405 DMRs found, only 3,850 were distributed within the promoter region, indicating that the majority of the DMRs are distributed within the gene body and intergenic regions. The DNA methylation of the gene body is complex, and a number of studies have demonstrated that methylation in this region can negatively regulate gene expression (Hu et al., 2013; Long et al., 2014). However, a number of studies suggest that it does not inhibit, but promote, gene expression (Yang et al., 1999; Ball et al., 2009; Yuan et al., 2016), even though some studies in plants have shown that DNA methylation in the gene body region may not regulate gene expression (Bewick and Schmitz, 2017), so the effect of methylation in the gene body and intergenic regions remains controversial (Flanagan and Wild, 2007). In this study, the mRNA expression of the seven DMGs annotated in the promoter region was analyzed by qRT-PCR. The results suggest that methylation in the promoter region negatively regulates gene transcription. The methylations in the promoter regions regulate gene transcription based on inhibition of transcription factor binding. These methylation sites in DMGs can be used as candidate epigenetic biomarkers of dilution phenotypes.

In the present study, DMGs involved in the regulation of pigmentation were identified by GO and KEGG enrichment analysis, including ASIP, MITF, RAB27A, MYO5A, MLPH, SLC36A1, SOX10, TYR, TYRP1, PMEL, WNT5B, USP13, SLC7A11, etc. Of these, it is known that the complexes of RAB27A and MYO5A can affect the capture, transport, and distribution of melanosomes and hence pigmentation (Fukuda et al., 2002). The agouti and extension loci which encode ASIP and MC1R genes can affect mammalian pigmentation by regulating the relative quantity of two melanin types (Bultman et al., 1992; Robbins et al., 1993). SLC36A1 helps regulate intracellular pH by participating in the maturation of melanin bodies, and its missense mutation in exon 2 is responsible for the champagne dilution in horses (Cook et al., 2008). MITF regulates differentiation and development in melanocytes, while its stability is regulated by the USP13 deubiquitinase (Shibahara et al., 2001; Zhao et al., 2011). SLC7A11 is involved in pigmentation by decreasing melanocyte apoptosis and modulated by POU2F1 (Chen et al., 2019). In this study, these DMGs were mostly located in gene body regions of the genome. The manner in which methylation affects the regulation of gene function in DMGs requires further investigation. Moreover, how the methylation modification of these DMGs is involved in coat color dilution of rabbits will be the focus of our next study, and the sample size of WGBS in this study was limited. The conversion efficiency of disulfite-treated DNA determines the accuracy of DNA methylation detection, and incomplete DNA transformation will lead to false positive results in the analysis, so replication of the results with a larger sample size would be important in future studies.

## Conclusions

In summary, we have investigated the global DNA methylation pattern of rabbit hair follicles associated with standard Chinchilla and diluted Chinchilla groups. A number of related DMRs were revealed to contribute to our understanding of the epigenetic regulation of rabbit inherited coat color dilutions.

## Data Availability Statement

WGBS data were submitted to National Center for Biotechnology Information (NCBI) Sequence Read Archive (SRA) database under the accession number SRP107834. Other datasets can be found in FigShare - https://figshare.com/, 10.6084/m9.figshare.12924452.

## Ethics Statement

The animal study was reviewed and approved by the recommendations of Animal Care and Use Committee at Yangzhou University. Written informed consent was obtained from the owners for the participation of their animals in this study.

## Author Contributions

YC was responsible for the collection and analysis of results and wrote the manuscript. YC, SH, ML, BZ, NY, and JL performed experiments. QC, JZ, and GB prepared figures and/or tables. YC and XW designed the study. All authors contributed to the article and approved the submitted version.

## Conflict of Interest

The authors declare that the research was conducted in the absence of any commercial or financial relationships that could be construed as a potential conflict of interest.

## Abbreviations

Ch: Chinchilla group
DCh: diluted Chinchilla group
DMRs: differentially methylated regions
DMGs: DMR-associated genes
GO: Gene Ontology
KEGG: Kyoto Encyclopedia of Genes and Genomes
BSP: bisulfite sequencing PCR
LTR: long terminal repeat
TSS: transcription start site.
