# Supplementary material for: Analysis of Genome DNA Methylation at Inherited Coat Color Dilutions of Rex Rabbits
Source: Front Genet. 2021 Jan 21;11:603528. doi: 10.3389/fgene.2020.603528 (PMC7859435; doi:10.3389/fgene.2020.603528)
Supplement: Supplementary file 1 [file Table_1.DOCX]

**Table S1** Allele frequency and genotype frequency of MLPH in diluted Chinchilla population

| Exon | SNPs | color | genotype frequency | |  | Allele frequency | | He | χ2 | PIC | HW |
| --- | --- | --- | --- | --- | --- | --- | --- | --- | --- | --- | --- |
| 5 |  |  | CC | CA | AA | C | A |  |  |  |  |
|  | g.606C>A | DCh | 0.45 | 0.55 | 0 | 0.725 | 0.275 | 0.399 | 0.2 | 0.319 | 2.878 |
|  |  |  | TT | TC | CC | T | C |  |  |  |  |
|  | g.610T>C | DCh | 0.3 | 0.35 | 0.35 | 0.475 | 0.525 | 0.499 | 0.1 | 0.374 | 1.779 |
|  |  |  | CC | CG | GG | C | G |  |  |  |  |
|  | g.642C>G | DCh | 0.25 | 0.35 | 0.4 | 0.425 | 0.575 | 0.489 | 0.7 | 0.369 | 1.611 |
| 8 |  |  | AA | AG | GG | A | G |  |  |  |  |
|  | g.894A>G | DCh | 0.25 | 0.45 | 0.3 | 0.475 | 0.525 | 0.499 | 1.3 | 0.374 | 0.191 |
|  |  |  | TT | TC | CC | T | C |  |  |  |  |
|  | g.905T>C | DCh | 0.25 | 0.45 | 0.3 | 0.475 | 0.525 | 0.499 | 1.3 | 0.375 | 0.191 |
|  |  |  | GG | GC | CC | G | C |  |  |  |  |
|  | g.941G>C | DCh | 0.3 | 0.45 | 0.25 | 0.525 | 0.475 | 0.499 | 1.3 | 0.374 | 0.191 |
|  |  |  | AA | AG | GG | A | G |  |  |  |  |
|  | g.953T>C | DCh | 0.25 | 0.45 | 0.3 | 0.475 | 0.525 | 0.499 | 1.3 | 0.374 | 0.191 |
| 9 |  |  | AA | AG | GG | A | G |  |  |  |  |
|  | g.1067A>G | DCh | 0.35 | 0.35 | 0.3 | 0.525 | 0.475 | 0.499 | 0.1 | 0.374 | 1.779 |
|  |  |  | CC | CT | TT | C | T |  |  |  |  |
|  | g.1095C>T | DCh | 0.35 | 0.35 | 0.3 | 0.525 | 0.475 | 0.499 | 0.1 | 0.374 | 1.779 |
| 12 |  |  | CC | CG | GG | C | G |  |  |  |  |
|  | g.1462G>A | DCh | 0.35 | 0.4 | 0.25 | 0.55 | 0.45 | 0.495 | 0.7 | 0.372 | 0.737 |
|  |  |  | GG | GA | AA | G | A |  |  |  |  |
|  | g.1462G>A | DCh | 0.35 | 0.4 | 0.25 | 0.55 | 0.45 | 0.495 | 0.7 | 0.372 | 0.737 |
|  |  |  | AA | AG | GG | A | G |  |  |  |  |
|  | g.1463A>G | DCh | 0.35 | 0.4 | 0.25 | 0.55 | 0.45 | 0.495 | 0.7 | 0.372 | 0.737 |
|  |  |  | AA | AG | GG | A | G |  |  |  |  |
|  | g.1482A>G | DCh | 0.35 | 0.4 | 0.25 | 0.55 | 0.45 | 0.495 | 0.7 | 0.372 | 0.737 |

*The polymorphism results of MLPH in Ch group have been published（Li et al., 2020).*
