# Supplementary material for: Analysis of Genome DNA Methylation at Inherited Coat Color Dilutions of Rex Rabbits
Source: Front Genet. 2021 Jan 21;11:603528. doi: 10.3389/fgene.2020.603528 (PMC7859435; doi:10.3389/fgene.2020.603528)
Supplement: Supplementary file 2 [file Table_2.DOCX]

**Table S2** BSP primer sequences

| Gene | Sequence (5′ → 3′) | length | Tm | Region |
| --- | --- | --- | --- | --- |
| USP13 | F:AATGTTTAGTATGTTGAAAGAGATTAT  R: AAATTTATAAACAAAACTCCAAAC | 359bp | 55°C | intron |
| PKP2 | F:AATGATGTAGTTTTAAAAGTATGTTAGAGA  R: CCATAAAATACAAAACCCAAACACT | 301bp | 55°C | intron |
