# Supplementary material for: Analysis of Genome DNA Methylation at Inherited Coat Color Dilutions of Rex Rabbits
Source: Front Genet. 2021 Jan 21;11:603528. doi: 10.3389/fgene.2020.603528 (PMC7859435; doi:10.3389/fgene.2020.603528)
Supplement: Supplementary file 3 [file Table_3.DOCX]

**Table S3** RT-PCR primer sequences

| Primer | Sequence (5′ → 3′) | Product length (bp) |
| --- | --- | --- |
| DCT | Forward: ATTCTGCTGCCAATGACCC | 154 |
|  | Reverse: AACGGCACCATGTTATACCTG |  |
| TCF7L1 | Forward: CACGGCAGCATGTTGGACT | 114 |
|  | Reverse: TGGCTTGGTGGTGAGGGAC |  |
| SZT2 | Forward: CCACCTGTCTTCGCCAAACTC | 136 |
|  | Reverse: AAGAAGCCACTGTCAGGGTCC |  |
| ARAF | Forward: GTCTATGCCTACGGGGTTGTG | 143 |
|  | Reverse: GGGCAGTTGCTGGAGATTTTA |  |
| GSTA4 | Forward: CCGAAGCATCCTCCATT | 103 |
|  | Reverse: GAGATCCAGCGTCCCCT |  |
| EDA | Forward: CACTCTCCCCCGACTCCC | 100 |
|  | Reverse: CGAACACGCCTACTTTCCTCT |  |
| WNT10A | Forward: GTCACCCGACTTCTGCGAG | 139 |
|  | Reverse: GGTCTGGCGTAGGATGTTGT |  |
| GAPDH | Forward: CACCAGGGCTGCTTTTAACTCT | 141 |
|  | Reverse: CTTCCCGTTCTCAGCCTTGACC |  |
